# Transient Impact Response of Plates Containing Flaws

**DOI:** 10.6028/jres.092.038

**Published:** 1987-12-01

**Authors:** Mary Sansalone, Nicholas J. Carino

**Affiliations:** National Bureau of Standards Gaithersburg, MD 20899

**Keywords:** finite element analysis, flaw, frequency spectrum analysis, impact, impact-echo method, nondestructive testing, stress wave propagation

## Abstract

The finite element method was used to study the transient response to point impact of thick circular plates containing disk-shaped flaws. The response was studied in both the time and the frequency domains, and compared to the response obtained from a solid plate. The effects on the response caused by changing the diameter and depth of a flaw, the duration of the impact, and the position where the response is calculated were determined. From the results of these parameter studies, conclusions were drawn which can be used in planning and interpreting impact-echo laboratory and field test results.

## Introduction

The results of axisymmetric, finite element studies of the transient response of thick, circular plates containing planar disk-shaped flaws subjected to point impact are presented in this paper. The capability of the finite element method for modeling transient stress wave propagation in elastic solids containing flaws was established in previous papers by the authors [1–4].

These studies are part of an ongoing research project at the National Bureau of Standards (NBS) which is aimed at developing a basis for a nondestructive test method for flaw detection in heterogenous materials, such as concrete, using transient stress waves [1,5,6]. The experimental technique (impact-echo method) involves introducing a transient stress pulse into a test object by mechanical impact at a point. At an adjacent point, the surface displacement produced by the arrival of reflections of the pulse from internal defects and external boundaries is monitored and the recorded waveforms are used to obtain information about the interior of a test object. Frequency spectrum analysis of waveforms is used to facilitate signal interpretation [1,6,7].

**Figure 1:**
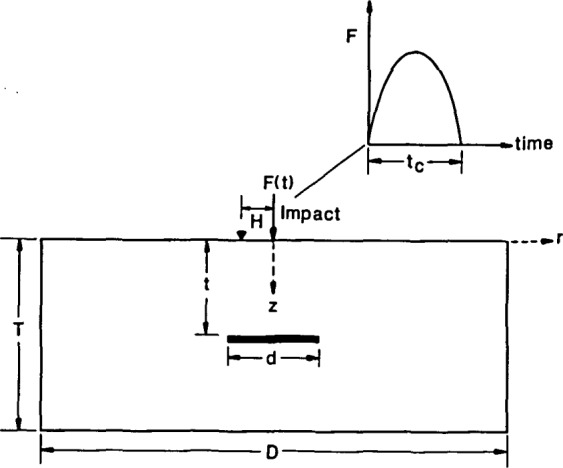
Schematic representation of point impact on a circular plate containing a disk-shaped void.

The objective of this study is to determine the effect of test variables on the ability to discern the presence of disk-shaped voids within plates from displacement waveforms and their frequency spectra. Figure 1 shows a schematic representation of point impact on a circular plate containing a disk-shaped void. A suitable point impact is generated by dropping a small steel sphere onto the top surface of the plate; the time-history of the contact force due to the elastic impact was approximated as a half-cycle sine curve (Goldsmith).

The important variables affecting the response of the flawed plate are: the diameter, *D*, and thickness, *T*, of the plate *(DIT* aspect ratio); the diameter, *d*, and depth, *t*, of the flaw; the frequency content of the stress pulse generated by impact; and, the distance, *H*, from the impact point to the point where the response is monitored. A study of the transient responses of unflawed circular plates of various geometries was presented in a previous paper [4]. In this study only circular plates with a diameter of 2 m and a thickness of 0.5 m were analyzed. The effect of the other variables on displacement waveforms and spectra will be discussed in this paper. Finally, it will be shown that the length of time the response is monitored, that is, the record length, is also an important factor.

## Solid Plate Response

In this section, the impact response of a solid plate obtained from a finite element analysis is discussed to establish a basis for comparison to responses obtained from flawed plates. For this analysis, the elastic properties of the plate were a modulus of elasticity of 33100 MPa and a Poisson’s ratio of 0.2. The density was 2300 kg/m^3^. These properties resulted in P-, S-, and R-wave speeds of 4000, 2440, and 2240 *m/s*, respectively. The impact was simulated as a pressure load over a 0.005-m diameter region at the top center of the circular plate. The time-history of the pressure was a half-cycle sine curve with a duration (contact time) of 40 microseconds.

**Figure 2:**
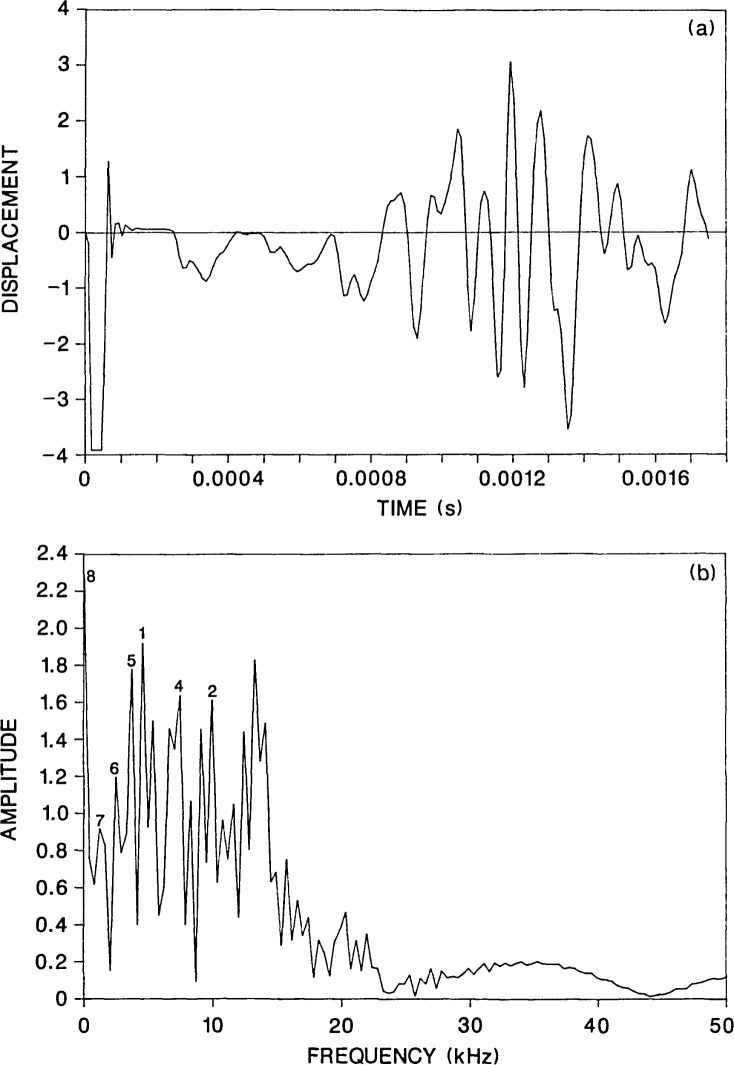
Finite element response of a point on the top surface of a solid 0.5-m thick, 2-m diameter plate subjected to a 40-microsecond duration impact: a) normal displacement waveform (*H* =0.03 m); and, b) spectrum.

Figure 2(a) shows a displacement waveform obtained at a point located a distance of 0.03 m from the impact. The response consists of the following components: an initial large downward displacement caused by the Rayleigh (R) wave arrival; displacements caused by the arrival of P- and S-waves which have been multiply reflected or mode-converted between the top and bottom surfaces of the plate (e.g., the arrival of the first P-wave reflected from the bottom surface is labeled by a 2P); and finally, displacements caused by the arrival of waves reflected and mode-converted from the perimeter of the plate, which are superimposed upon the displacements caused by waves multiply reflected between the top and bottom surfaces of the plate. The R-wave has been clipped, as is normally done in laboratory studies, to accentuate the displacement caused by body waves [1,7].

Figure 2(b) shows the amplitude spectrum obtained by taking the Fast Fourier Transform of the waveform shown in figure 2(a). The waveform contained 256 points and the sampling interval was 9.4 microseconds; thus, the interval in the spectrum is 0.42 kHz. For the 40 microsecond duration impact, most of the energy in the stress waves is contained in frequencies of 37 kHz or less. This value is obtained from the value of the first zero that occurs in the spectrum of the impact force-time function [7]. Note also that before the Fast Fourier Transform of a waveform was computed, it was shifted to remove the component of the displacement caused by rigid body translation of an unsupported plate subjected to impact [4]. This shift significantly reduced the zero frequency component in the spectrum.

The spectra obtained from thick circular plates subjected to point impact exhibit peaks at resonant frequencies corresponding to the following modes [4]: waves propagating between the top and bottom surfaces of the plate (thickness modes, Nos. 5 and 6); waves propagating across the diameter of the plate (diameter modes, Nos. 7 and 8); and, flexural modes (Nos. 1–3) and the rod mode (No. 4). For each of these modes, the calculated values and the digital values from the finite element analysis are listed in table 1. All of these modes are present in figure 2(b) giving rise to a complicated response spectrum.

In the following discussion, it is shown how the presence of a disk-shaped void within the plate, depending upon its size and location, can dramatically alter the solid plate response. In all of the cases discussed in the following sections, the length and sampling interval of the time domain records, and the dimensions and material properties of the plates containing the flaws are the same as for the solid plate.

## Response of Plates Containing Flaws

**Figure 3:**
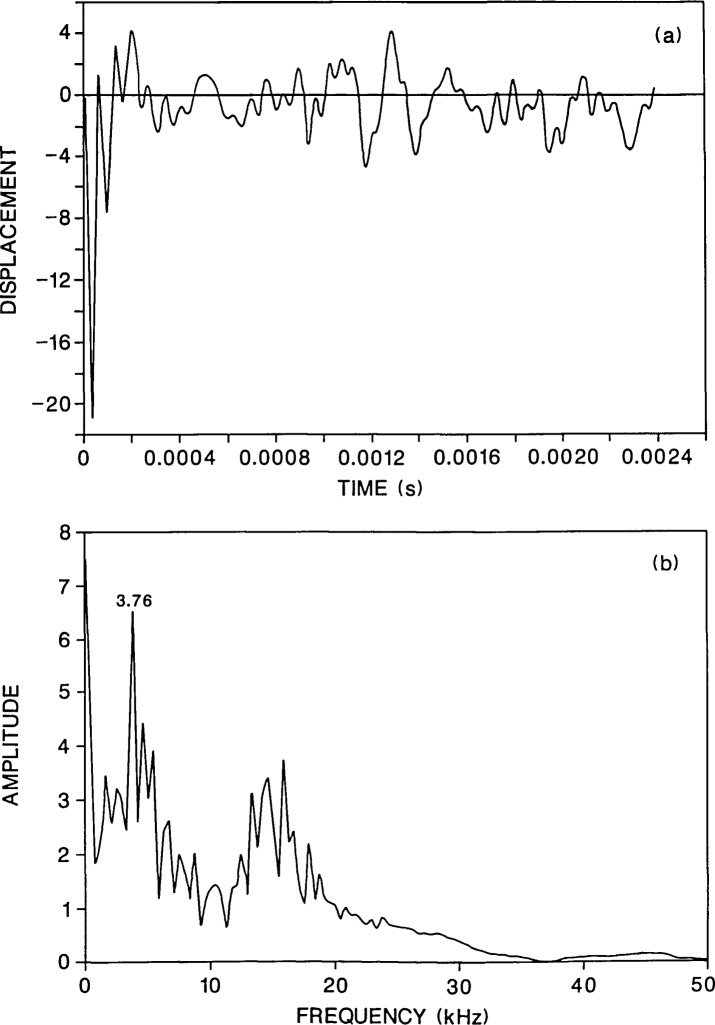
Finite element response of a point on the top surface of a plate containing a 0.2-m diameter flaw located 0.13 m below the top surface. The plate was subjected to a 40-microsecond duration impact: a) displacement waveform (*H* =0.03 m); and, b) spectrum.

Figure 3(a) shows the calculated response for a plate containing a 0.2-m diameter flaw located 0.13 m below the top surface of the plate. The flaw is a 0.01-m thick disk-shaped void. Since the problem is axisymmetric, the center of the flaw coincides with the center of the plate and the impact point is directly above the center of the flaw. The displacement was calculated 0.03 m from the impact point so that a direct comparison could be made with the solid plate results (fig. 2). The waveform for the flawed plate [fig. 3(a)] is very different from the response of the solid plate [fig. 2 (a)] because it contains high frequency oscillations superimposed upon the plate response. These oscillations are caused by P-wave reflections between the flaw and the top surface of the plate^1^.

Figure 3(b) shows the spectrum of the waveform in figure 3(a). There is a large amplitude peak at 3.76 kHz which corresponds to the frequency of P-waves that have diffracted around the edge of the flaw and been reflected from the bottom surface of the plate. (The reader is referred to refs. [1 and 3] which present detailed discussions of diffraction at the edge of a flaw.) This peak is the same P-wave thickness mode peak that was present in figure 2(b); however, in figure 3(b) the 3.76-kHz peak has a much larger amplitude compared with the remainder of the peaks in the spectrum. In figure 3(b) there is also a group of large amplitude peaks near 15 kHz. These peaks are caused by P-wave reflections from the flaw. For a P-wave speed of *C*_P_ and a flaw depth, *t*, the thickness mode frequency for P-waves is:

$$f=C_{P}/(2t),$$ (1)

which in this case is 15.4 kHz. Thus, reflections from the flaw produce series of frequency peaks clustered around the thickness mode frequency. In this case, the depth, *t*, of the flaw is known. If however, the depth is not known, it can be calculated from the peak frequency [eq (1)]. If the high amplitude peak at 15.9 kHz is used in equation (1), the computed depth is 0.126 m which is close to the exact depth of 0.13 m. Note that equation (1) is valid for responses measured close to the impact point.

An interesting result that appears in figure 3(b) is that the frequency peaks which are caused by resonances set up by the plate perimeter are of secondary importance [compare with fig. 2(b)]. This result is advantageous in terms of flaw detection as it allows the presence of the flaw to be easily detected and the depth of the flaw to be accurately determined.

A convenient parameter that will be used in comparisons of flaw geometries is the diameter to depth (*d*/*t*) ratio of the flaw. In this case, the presence of a flaw with a *d*/*t* ratio of 1.54 significantly altered the plate response. Before presenting results obtained from other flaw geometries, the effect of changing the contact time of the impact and of changing the location where the displacement is recorded will be discussed.

## Contact Time of the Impact

The frequency content of the stress pulse produced by impact depends on the contact time of the impact [7]. A shorter contact time produces a pulse which contains energy in a broad frequency range. A longer contact time produces a pulse which primarily contains large amplitude, low frequency components. This is important because the frequency content of the waves produced by impact determines the size of the flaw that can be detected. For a flaw to be detected, it is generally stated that the dimensions of the flaw must be on the order of, or larger than, the component wavelengths in the propagating waves. For example, to detect a 0.2-m diameter flaw in concrete, frequencies in the range of 20 kHz or higher would probably be needed.

In impact-echo testing of concrete, 40- to 80-microsecond duration impacts are typical values produced by the impact of small diameter (7–12 mm) steel spheres. Figure 3 shows the response for a plate subjected to a 40-microsecond duration impact. In this case, the depth of the flaw was easily determined as a significant portion of the input energy was contained in frequencies greater than 20 kHz.

**Figure 4:**
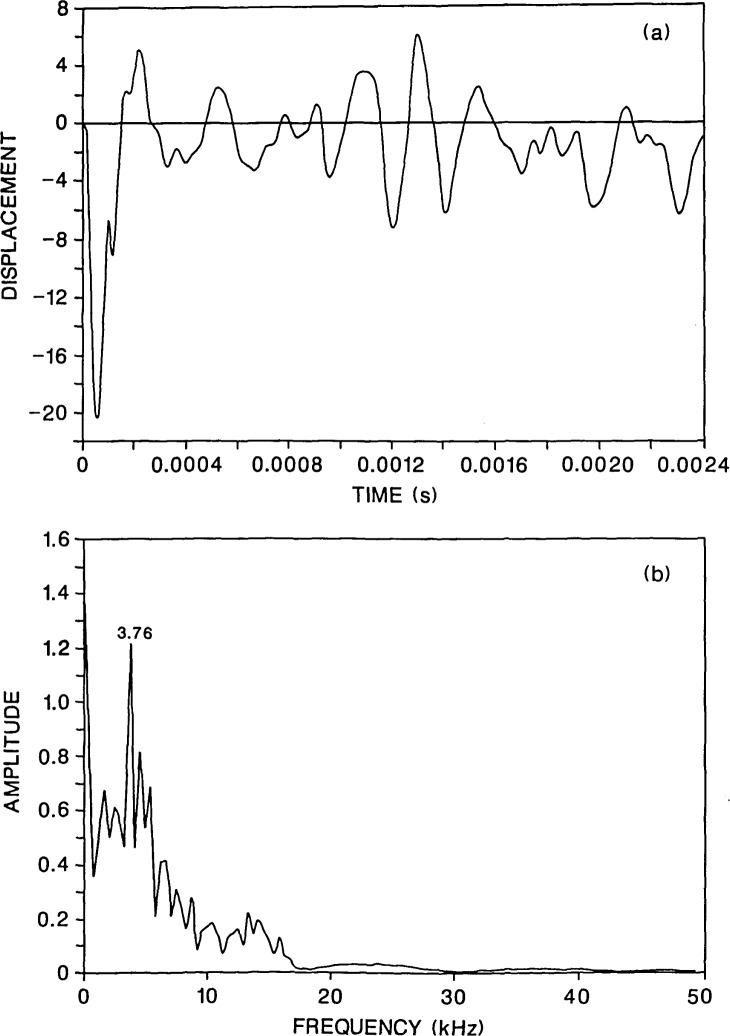
Finite element response of a point on the top surface of a plate containing a 0.2-m diameter flaw located 0.13 m deep. The plate was subjected to a 80-microsecond duration impact: a) displacement waveform (*H*=0.03 m); and, b) spectrum.

For comparison, figure 4 shows the response at the same point for an 80-microsecond duration impact on the same plate. For this contact time, most of the energy in the stress waves produced by the impact is contained in frequencies less than approximately 19 kHz. Thus, only a small amount of energy will be reflected by the flaw. The waveform in figure 4(a) does not contain the high frequency oscillations that are present in figure 3(a). In addition, in the spectrum in figure 4(b), the peaks near 15 kHz are of very low amplitude relative to the P-wave plate thickness peak at 3.76 kHz. The depth of the flaw would be difficult to determine, however, the flaw would probably be detected, because the waveform and spectrum looks very different from that obtained from the solid plate (fig. 2). In figure 4(a), the portion of the waveform between the R-wave and the arrival of reflections from the side of the plate shows displacements which are well above the zero displacement line. These upward displacements are caused by waves diffracted from the edge of the flaw [1,3]; they do not occur in the solid plate response [fig. 2(a)]. Also, the spectrum in figure 4(b) does not exhibit all the various plate modes that are present in the solid plate response [fig. 2(b)].

Thus the contact time of the impact is an important factor in impact-echo testing for detection of flaws. The contact time must be chosen based on the material properties of the structure being evaluated and the size of flaw to be detected. For example, longer duration impacts must be used to penetrate heterogenous solids such as concrete and this limits the minimum size of flaw that can be detected. These results also show that the presence of a flaw will have an effect on the plate response even if the flaw cannot be accurately located using a given impact. It should be noted that the success of the impact-echo method in laboratory and field test conditions relies on having a high fidelity transducer capable of measuring surface displacements. A transducer which has been used successfully is the conical transducer developed at NBS [10].

## Location Where Response is Recorded

Surface displacement waveforms are also affected by: 1) the distance, *H*, between the impact point and the point where the displacement is recorded; and, 2) the location of the point where the displacement is recorded relative to the location of the flaw. It seems obvious to expect that the displacement response will be different if it is measured close to the impact point and directly over the central region of the flaw as compared to being recorded at a point that is over or off the edge of the flaw.

**Figure 5:**
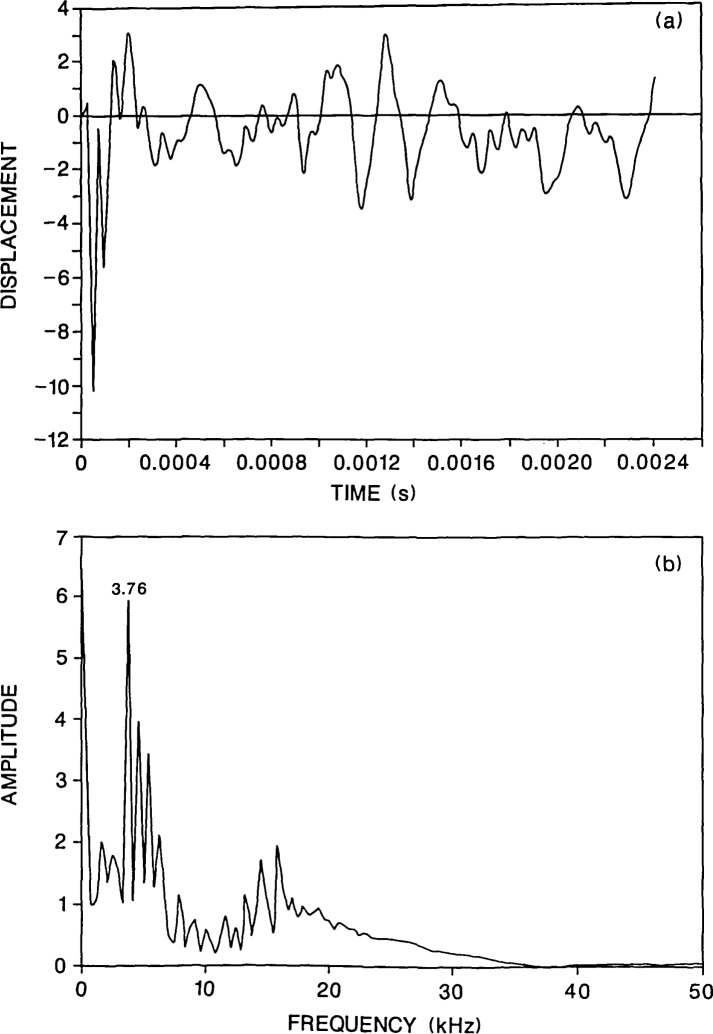
Finite element response of a point on the top surface of a plate containing a 0.2-m diameter flaw located 0.13 m deep: a) displacement waveform (*H*=0.06 m); and, b) spectrum.

**Figure 6:**
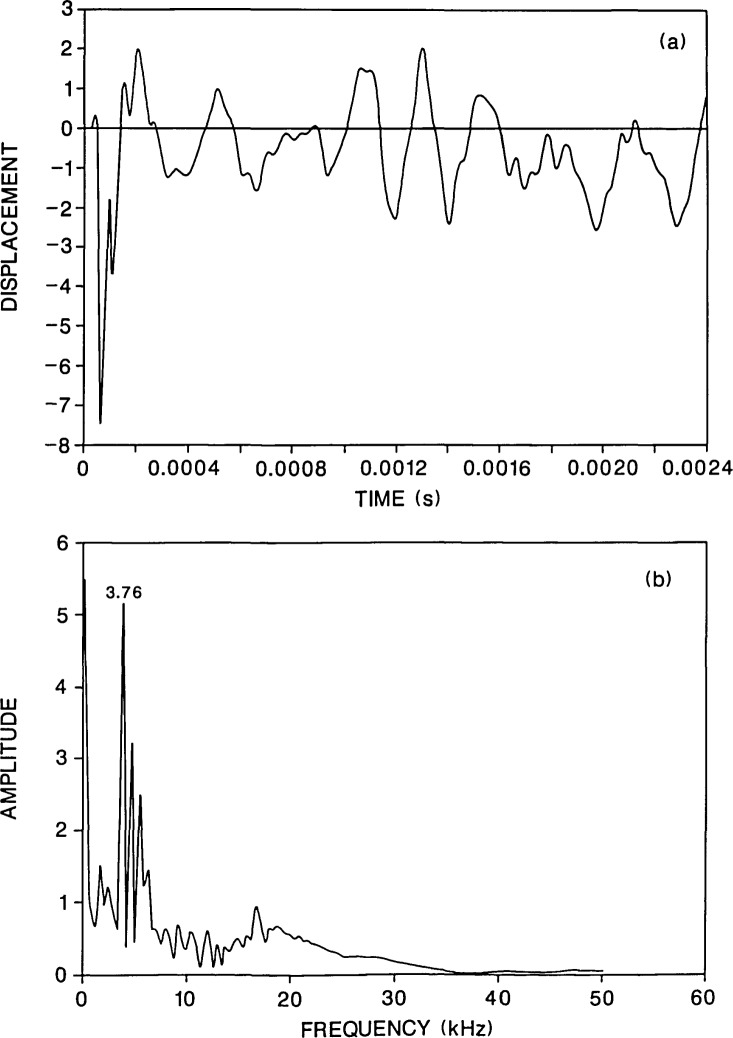
Finite element response of a point on the top surface of a plate containing a 0.2-m diameter flaw located 0.13 m deep: a) displacement waveform (*H* = 0.09 m); and, b) spectrum.

Figures 5 and 6 show waveforms and their corresponding spectra for the plate containing the 0.2-m diameter flaw located 0.13 m deep for values of *H* equal to 0.06 and 0.09 m, respectively. The duration of the impact was 40 microseconds. A comparison of the results shown in figures 5 and 6 with those shown in figure 3 (*H* =0.03 m), shows that as *H* increases, the high frequency oscillations due to P-wave reflections from the flaw diminish in amplitude in the waveform and there is a corresponding decrease in the amplitude of the peaks near 15 kHz in the frequency spectra. In contrast, the frequency peak produced by multiple reflections of the P-wave between the top and bottom surfaces of the plate shows only a slight decrease in amplitude.

In figures 3 and 5, the presence of the flaw can be easily detected in the waveforms and in the spectra. In figure 6, the case where the response point is nearly above the edge of the flaw, the peaks in the spectrum near 15 kHz are much lower in amplitude; however, the approximate depth of the flaw can still be determined from the single peak at 16.7 kHz. In figure 6, the presence of a flaw can also be discerned for the two reasons discussed previously: the spectrum looks very different from that shown in figure 2 for a solid plate; and, the initial portion of the displacement waveform exhibits both high frequency oscillations and displacements that are above the zero line, indicating the presence of a sharp edge causing diffraction.

In summary, these results show that it is best to record the response near the impact point. This assures that if the impact point is over a flaw, then the maximum response caused by P-waves reflected from the flaw will be recorded and the flaw will be easier to detect and its depth easier to determine.

The discussion up to this point has focused on a plate containing a 0.2-m diameter flaw at a depth of 0.13 m—a flaw geometry with a large *d*/*t* ratio of 1.54. In the following section, the effect of changing flaw size and flaw depth will be shown.

## Flaw Diameter and Flaw Depth

**Figure 7:**
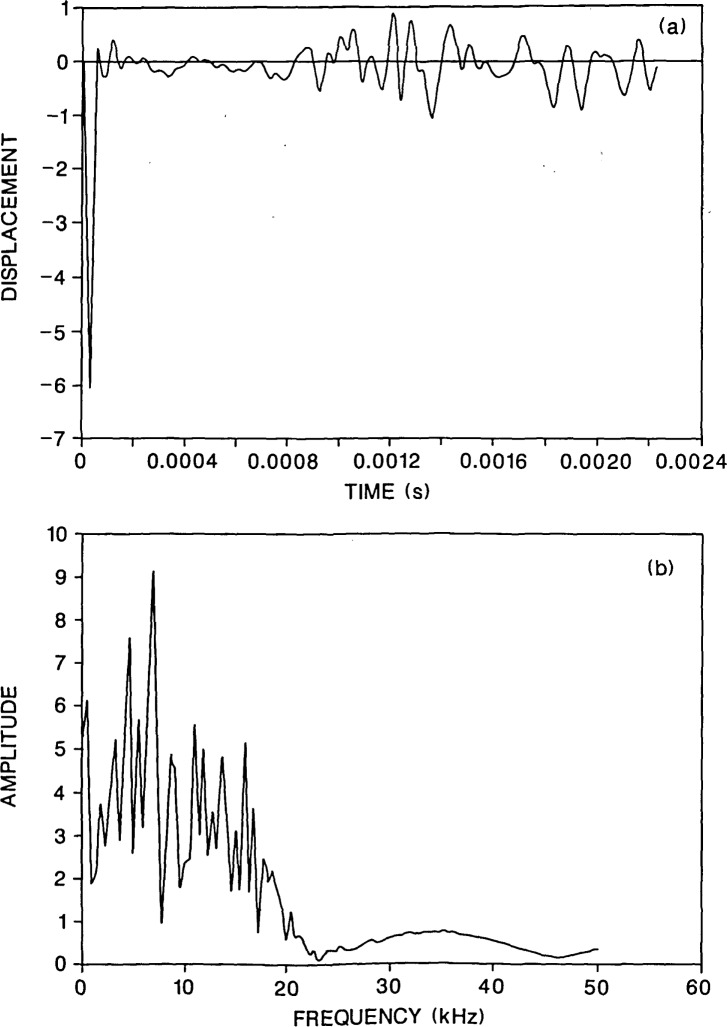
Finite element response of a point on the top surface of a plate containing a 0.07-m diameter flaw located 0.13 m deep: a) displacement waveform (*H* =0.03 m); and. b) spectrum.

**Figure 8:**
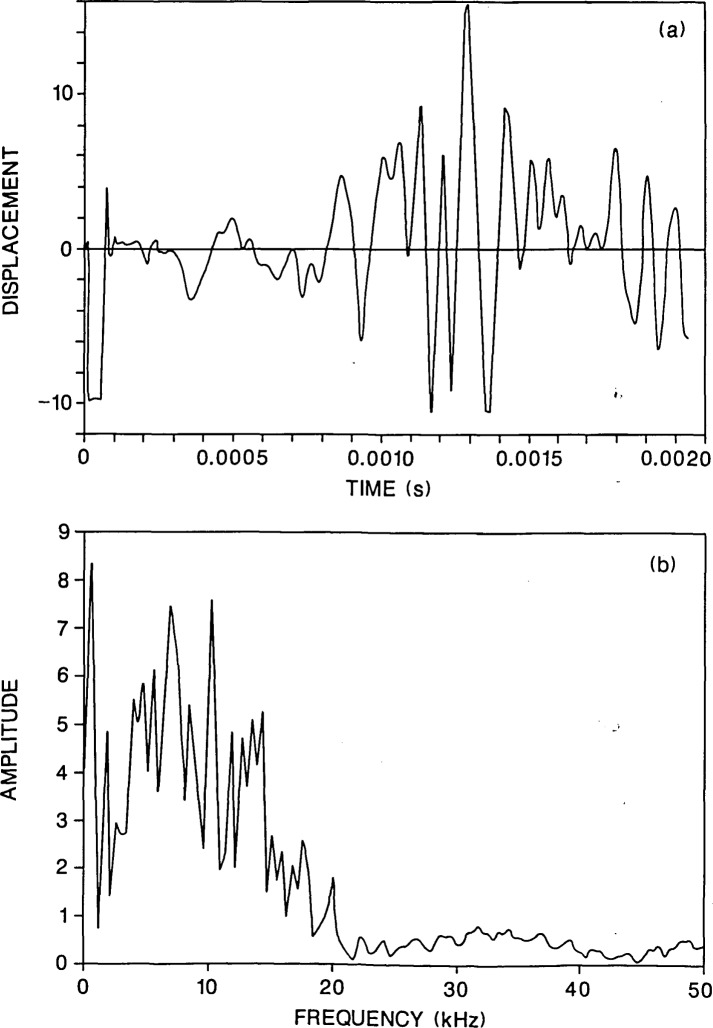
Finite element response of a point on the top surface of a plate containing a 0.03-m diameter flaw located 0.38 m deep: a) displacement waveform (*H* =0.03 m); and, b) spectrum.

To show the effect of changing flaw diameter and depth, results of two analyses will be presented: 1) a plate containing a 0.07-m diameter flaw located 0.13 m below the surface of the plate (fig. 7); and 2) a plate containing a 0.2-m diameter flaw located 0.38 m below the surface of the plate (fig. 8). Both cases result in *d/t* values of 0.53. The duration of the impact was 40 microseconds and the displacement was calculated 0.03 m from the impact point. Thus a direct comparison can be made with the results shown in figures 2 and 3.

The initial portions of the waveforms in figures 7(a) and 8(a) show oscillations and displacements which are absent in the solid plate response [fig. 2(a)]. Therefore, the waveforms reveal that a flaw exists in both plates. Figures 7(b) and 8(b) show the frequency spectra of these waveforms; these spectra are very different from the spectrum shown in figure 3(b). In fact, they resemble the solid plate spectrum [fig. 2(b)], exhibiting a myriad of peaks corresponding to all the modal plate vibrations and resonances set up by waves propagating in the plate. However, there are differences in the frequency spectra for the plates containing the flaws as compared with the spectrum for the solid plate; these differences give indications of the presence of a flaw, but probably would go unnoticed in actual testing where the presence of a flaw was not known beforehand. In figure 7(b), there is a larger amplitude peak (as compared with figure 2(b) at 15.8 kHz which is close to the P-wave peak produced by reflections from the surface of the flaw, but this is also the frequency of the third flexural mode of the plate. Therefore, if flaw detection were based solely on the spectrum, the flaw would probably not be noticed. In figure 8(b), there is a secondary peak at 5.4 kHz which is the frequency of P-wave reflections from the surface of the flaw, but this peak is also present in figure 2(b); again if only the spectrum is used, the flaw would not be detected. In these cases, the spectra are of no help in flaw detection; however, looking at the waveforms, the effects caused by the flaws are obviously present if the displacement responses are compared to the solid plate response. As mentioned previously, in actual laboratory test conditions, this type of signal interpretation relies on having a high fidelity displacement transducer.

## Record Length

**Figure 9:**
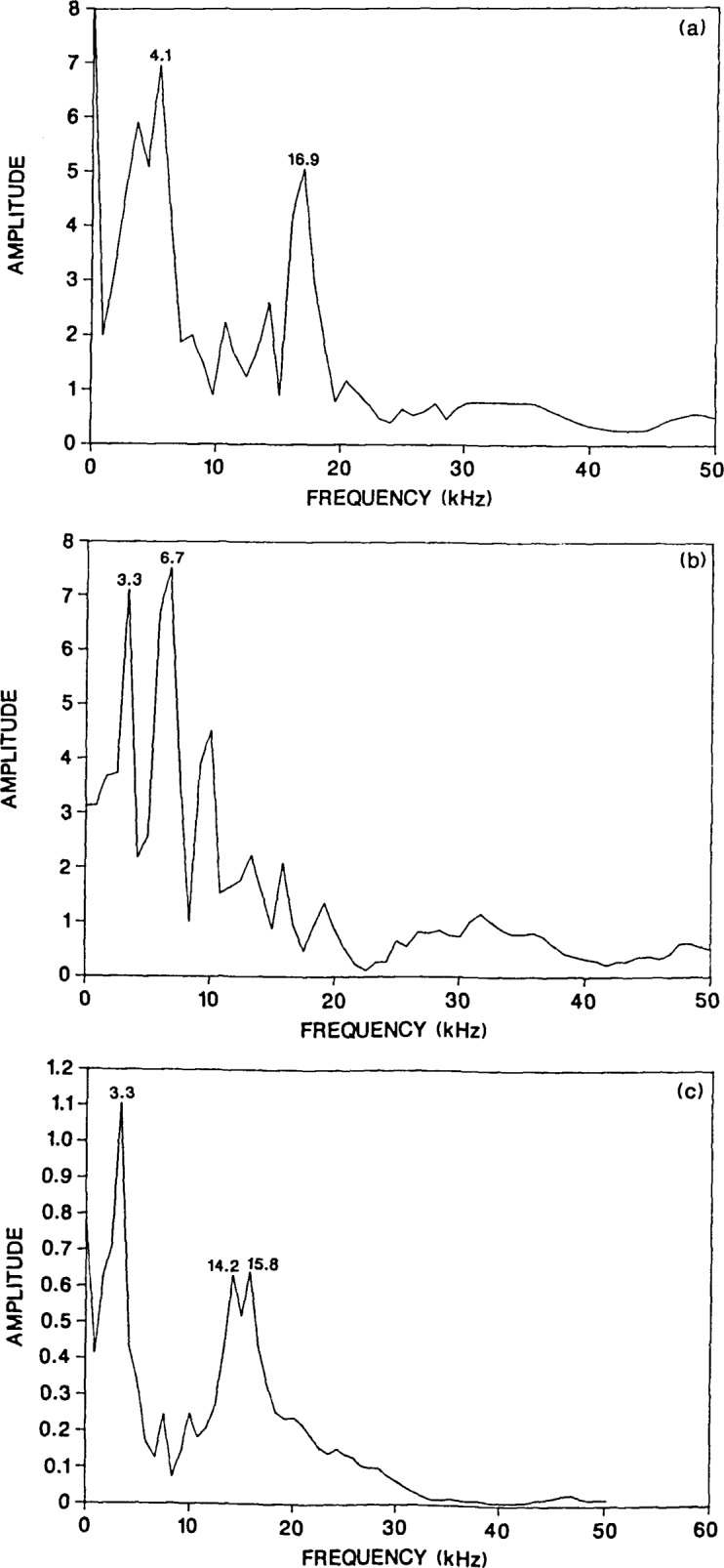
The effect of record length on frequency spectra. For a 1200 microsecond record length spectra were obtained from surface displacement waveforms from plates containing flaws: a) 0.07-m diameter flaw located 0.13 m deep (fig. 7); b) 0.2-m diameter flaw located 0.38 m deep (fig. 8); and, c) 0.2-m diameter flaw located 0.13 m deep (fig. 3).

The effects caused by the flaws are most apparent in the early portion of the waveforms shown in figures 7 and 8. To determine the effect of record length, only the first 1200 microseconds (128 points) of each waveform was transformed. The resulting spectra are shown in figures 9(a) and 9(b). For comparison the spectrum for the first 1200 microseconds of figure 3(a) was also obtained [fig. 9(c)]. The frequency interval in each spectrum is 0.83 kHz.

By using only the first 1200 microseconds of the waveforms, most of the response caused by the arrival of reflections from the plate perimeter was eliminated, and the corresponding spectra clearly reveal the presence of the flaw. The spectra in figures 9(a) and (b) show the same pattern as that obtained from the flaw with the large *d*/*t* value [fig. 3(b). There is a large amplitude peak(s) near 4 kHz caused by P-wave reflections between the top and bottom surfaces of the plate, and a second large amplitude peak caused by P-wave reflections from the surface of the flaw. In all cases, the depth of the flaw can be accurately determined.

Thus in impact-echo testing it appears to be advantageous to restrict the time-domain waveform to reflections occurring prior to multiple reflections from the side boundaries of a structure—reflections which give rise to flexural vibrations and other resonances. This is especially true for detecting smaller flaws or larger flaws located deep within a structure (smaller *d*/*t* values). However, by using a shorter record length, there is an increase in the frequency interval of the digital spectrum which can lead to inaccuracy in determining the depth of a flaw.

In summary, the smaller the *d*/*t* value, the more difficult it is to detect a flaw. Additional studies [1] have shown that for a given flaw depth, *t*, the critical value of the flaw diameter, *d*, increases as the contact time of the impact increases. Thus these three variables are related.

## Conclusions

This paper has focused upon the surface displacement responses produced by point impact on thick circular plates containing disk-shaped flaws. The effects on displacement waveforms and frequency spectra caused by the following test variables were determined: contact time of the impact; distance between the impact point and the response point; flaw diameter and depth; and the length of the time domain record. The following conclusions were made:

1. The contact time of the impact must be short enough so that sufficient energy is contained in the range of frequencies that will be reflected by a flaw. As the contact time increases, the range of frequencies in the input pulse decreases; therefore, the minimum flaw size that can be detected increases.
2. The point where the displacement is recorded should be kept close to the impact point to obtain maximum displacements caused by P-wave reflections.
3. The ratio of flaw diameter to flaw depth is a useful parameter for determining when planar disk-shaped flaws are likely to be detected. For a given contact time, the larger the *d*/*t* value of a flaw, the easier it is to detect the flaw.
4. Spectra obtained from time domain displacement records that are terminated before the arrival of reflections from the perimeter of the plate are much simpler to interpret, and clearly reveal the presence of flaws with smaller *d*/*t* values which would be hidden in spectra obtained from longer records.

The results of this study help to provide a theoretical basis for using the impact-echo method for finding flaws within heterogenous solids. In addition, this paper also shows the usefulness of the finite element method as a powerful tool for solving problems of transient wave propagation in solids containing flaws.

## Figures and Tables

**Table 1:** Comparison of calculated frequencies with frequencies based on finite element (FE) analyses^a^ (kHz)

Mode No.	Mode	0.5-m Plate *D*/*T*=4	
		Calc	FE
1	1st Flex	4.3	4.7
2	2nd Flex	10	11.1
3	3rd Flex	16	–b
4	Rod	7.5	7.1
5	P Thickness	4.0	3.7
6	S Thickness	2.4	2.5
7	P Diameter	1.7	1.7
8	S Diameter	0.43	0.42

**Note:** Resolution in finite element spectra is 0.42 kHz.

a Theoretical values for flexural and rod modes were obtained from published analytical and experimental results [11,12].

b Frequency not identified in spectra obtained from finite element analyses.

## Footnotes

1 Note that in figure 3(a), the high amplitude peak immediately following the downward dip due to the R-wave is caused by an overshoot response of the finite element model. This overshoot is caused by the rapid change in displacements produced by the R-wave, but it is quickly dampled out using artificial viscosities (8,9).
